# Reciprocal risks of diabetes and sarcopenia in aging Chinese adults: A prospective cohort study using a semi-Markov multi-state framework

**DOI:** 10.1097/MD.0000000000049398

**Published:** 2026-06-26

**Authors:** Yirui Chen, Yinuo Qu, Siyi Kong, Tianyun Wang, Zhiyuan Liu, Huixia Ren, Kai Ma, Tieniu Zhao, Hongwu Wang, Mengyang Wang

**Affiliations:** a College of Public Health, Tianjin University of Traditional Chinese Medicine, Tianjin, China; b Affiliated Hospital of Shandong University of Traditional Chinese Medicine, Jinan, Shandong Province, China; c College of Nursing, Guangxi University of Chinese Medicine, Nanning, China; d College of Culture and Health Communication, Tianjin University of Traditional Chinese Medicine, Tianjin, China.

**Keywords:** cohort study, diabetes mellitus, Markov model, multi-state model, sarcopenia, trajectories

## Abstract

Type 2 diabetes mellitus and sarcopenia are increasingly recognized as interrelated syndromes sharing pathophysiological pathways, yet empirical evidence for their reciprocal, time-dependent relationship in aging populations remains limited. Conventional analytical approaches fail to capture the dynamic, trajectory-based nature of their coprogression because of restrictive assumptions regarding state transitions and sojourn time. We conducted a prospective cohort study using harmonized data from 2 nationally representative Chinese longitudinal surveys: the China Health and Retirement Longitudinal Study and the Chinese Longitudinal Healthy Longevity Survey, with follow-up spanning 2008–2018. A semi-Markov multi-state model was employed to estimate sojourn-time-dependent transition hazards across 5 clinically defined states: diabetes-free/sarcopenia-free, diabetes-only, sarcopenia-only, comorbid diabetes-sarcopenia, and death (absorbing state). All models adjusted for time-varying sociodemographic, lifestyle, and functional covariates and accounted for competing risks. Population attributable fractions were derived via counterfactual simulation. The semi-Markov model demonstrated superior fit over the Markov specification (global ΔAkaike information criterion = 316.4), confirming strong sojourn-time dependence in transition risks. Incident diabetes was associated with an 82% higher hazard of progressing to comorbid diabetes-sarcopenia (hazard ratio = 1.82, 95% confidence interval [CI] = 1.47–2.25), while incident sarcopenia conferred a 65% elevated risk of subsequent diabetes onset (hazard ratio = 1.65, 95% CI = 1.35–2.02). These bidirectional associations remained robust across multiple sensitivity analyses. Counterfactual modeling revealed that 38.2% (95% CI = 31.5%–44.1%) of new comorbid cases could be prevented by blocking diabetes→sarcopenia progression, and 32.6% (26.8%–38.0%) by interrupting sarcopenia→diabetes progression. Strikingly, 58.9% (53.4%–64.1%) of all 5-year mortality was attributable to entry into any chronic disease state. These findings suggest that diabetes and sarcopenia are dynamically and bidirectionally associated in aging Chinese adults. Integrated assessment of glycemic status and muscle health may help identify older adults at elevated risk of comorbidity and mortality. Further interventional studies are needed to determine whether dual-domain prevention strategies can modify these trajectories.

## 1. Introduction

Type 2 diabetes mellitus (T2D) and sarcopenia are common age-related conditions that contribute substantially to disability, frailty, and mortality in older adults.^[1,2]^ Although traditionally considered separate metabolic and musculoskeletal disorders, increasing evidence suggests that these 2 conditions may be closely interconnected.^[3–5]^ Diabetes may accelerate muscle loss through insulin resistance, chronic inflammation, and impaired protein metabolism, whereas sarcopenia may worsen glucose homeostasis by reducing skeletal muscle mass, the major site of insulin-mediated glucose disposal.^[6–9]^

Despite this biological plausibility, the temporal and bidirectional relationship between diabetes and sarcopenia remains insufficiently understood. Most previous studies have relied on cross-sectional designs or conventional survival models, which are limited in their ability to establish temporal ordering, account for intermediate disease states, or capture changes in transition risk over time.^[10–12]^ In particular, standard Cox models usually treat disease status as a fixed exposure and do not explicitly model transitions between healthy, single-disease, comorbid, and death states.^[5,13–15]^

Multi-state models provide a useful framework for describing chronic disease progression as a dynamic process. Compared with conventional Markov models, semi-Markov models allow transition hazards to depend on sojourn time, that is, the time already spent in the current health state.^[2,8,16–18]^ This feature is clinically relevant for chronic conditions such as diabetes and sarcopenia, where the risk of subsequent disease or death may increase with longer disease duration.

Therefore, using harmonized longitudinal data from the China Health and Retirement Longitudinal Study (CHARLS) and the Chinese Longitudinal Healthy Longevity Survey (CLHLS), we applied a semi-Markov multi-state model to examine the reciprocal relationship between diabetes and sarcopenia in aging Chinese adults.^[13,19–21]^ We aimed to estimate transition-specific hazards among healthy, diabetes-only, sarcopenia-only, comorbid, and death states; to compare the semi-Markov specification with a conventional Markov model; and to evaluate the population-level impact of key transition pathways.

## 2. Method

### 2.1. Definitions

Diabetes mellitus (DM) was defined as meeting at least 1 of the following criteria: fasting plasma glucose ≥126 mg/dL, glycated hemoglobin A1c ≥6.5%, self-reported physician diagnosis, or current use of hypoglycemic medications.^[21]^ Sarcopenia was assessed according to the Asian Working Group for Sarcopenia 2019 consensus criteria, which require the presence of low muscle mass plus either low muscle strength or low physical performance.^[22]^ Muscle strength was evaluated using handgrip strength measured with a Yuejian WL-1000 dynamometer (Nantong Yuejian Physical Measurement Instrument Co., Ltd.).^[23]^ Participants performed 2 trials with each hand, holding the dynamometer at a 90° angle, and the highest value from either hand was used (if 1 hand was unavailable, the maximum from the other hand was recorded). Low handgrip strength was defined as <28 kg for men and <18 kg for women.^[22]^ Appendicular skeletal muscle mass (ASM) was estimated using a validated anthropometric prediction equation derived for the Chinese population:


$$\begin{array}{c} \begin{array}{cc} ASM\;\;\;=\;\;\;0.193*weight\left(kg\right)\;\;\;+\;\;\;0.107*height\left(cm\right)\\ & \;\;\;-\;\;\;4.157*sex\left(male:1,female:2\right)\;\;\;-\;\;\;0.037*age\left(yr\right)\;\;\;-\;\;\;2.361 \end{array} \end{array}$$

where weight (kg) was measured using an Omron HN-286 scale (Omron Healthcare Co., Ltd.), height (cm) with a Seca 213 stadiometer (seca GmbH & Co. KG), and sex was coded as 1 for male and 0 for female.^[24–26]^ This equation has demonstrated strong agreement with dual-energy X-ray absorptiometry (DXA)-derived ASM in prior studies.^[27]^ Low muscle mass was defined as sex-specific ASM adjusted for height squared (ASM/height^2^) below the 20th percentile of the study population (<6.79 kg/m^2^ for men; <4.89 kg/m^2^ for women).^[25,26]^ Physical performance was assessed using 3 components: usual gait speed over a 2.5-m course (timed round-trip); a total of 5-time chair stand test (time to rise 5 times from a 47-cm chair with arms folded); and the short physical performance battery, which combines gait speed, chair stands, and balance tests (side-by-side, semi-tandem, and tandem stands, each held for 10 seconds), yielding a total score of 0 to 12. Per Asian Working Group for Sarcopenia 2019, low physical performance was defined as gait speed <1.0 m/s, chair stand time ≥12 seconds, or short physical performance battery score <9.^[22]^ Participants were classified as having sarcopenia if they exhibited low muscle mass in combination with either low muscle strength or low physical performance.

### 2.2. Data sample composition

This study leverages data from 2 nationally representative longitudinal surveys in China: the CHARLS (http://charls.pku.edu.cn) and the CLHLS (https://chads.nsd.pku.edu.cn/sjzx/sjxz/index.htm).^[28–30]^ CHARLS uses a multistage, stratified probability sampling design to collect comprehensive multidimensional data – including sociodemographic characteristics, chronic disease history, biomarkers, and psychological well-being – from middle-aged and older adults across China. The CLHLS, initiated in 1998, focuses specifically on the determinants of healthy longevity among the oldest-old, covering participants from 22 provinces with 6 waves of data collection completed between 2002 and 2018. Both studies received ethical approval from the Biomedical Ethics Committee of Peking University (CHARLS: IRB00001052-11015; CLHLS: IRB00001052-13074), with CLHLS also approved by the Campus Institutional Review Board of Duke University (Pro00062871). All participants provided written informed consent, and the study protocols adhered to the ethical principles of the 2024 revision of the Declaration of Helsinki.^[31]^

The large reduction from the initial pooled sample to the final analytical risk sets reflects the specific requirements of directional disease-trajectory modeling rather than simple loss to follow-up. The initial sample included all participants from CHARLS and CLHLS across the relevant survey waves, many of whom were not eligible for the present analysis because they lacked complete information on diabetes status, sarcopenia components, follow-up time, mortality status, or covariates required for multi-state modeling. In addition, participants who did not contribute an interpretable temporal sequence for either diabetes preceding sarcopenia or sarcopenia preceding diabetes were not included in the corresponding directional risk set. Thus, the final analytical sample represents participants or participant-wave observations with sufficient longitudinal information to define disease states and estimate transition-specific hazards, rather than a simple subset of all survey respondents.

**Figure 1.**
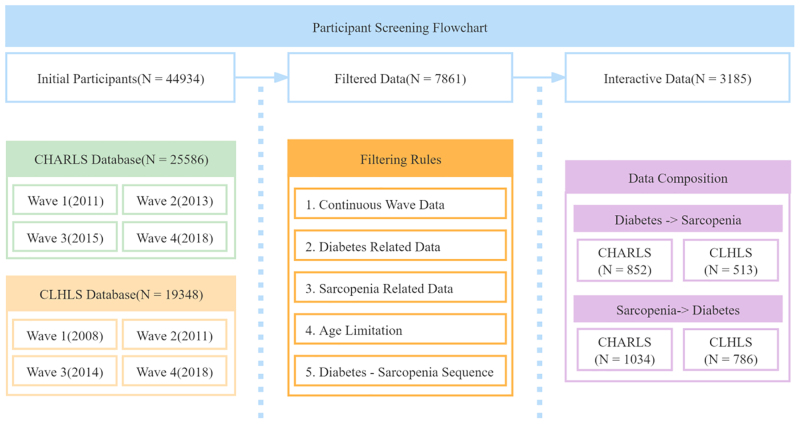
Participant screening flowchart.

This study was reported in accordance with the strengthening the reporting of observational studies in epidemiology guidelines.^[32]^ Figure 1 presents the participant screening flowchart. The initial pooled sample from the CHARLS and CLHLS surveys (2008–2018) comprised 44,934 individuals (25,586 from CHARLS and 19,348 from CLHLS). After applying stringent inclusion criteria – requiring complete data on diabetes and sarcopenia, a plausible temporal sequence between the conditions, and falling within the target age range – a total of 7861 individuals were eligible.

To explore the bidirectional relationship, we constructed 2 directional analytical subgroups from this eligible pool. This resulted in a final analytical sample of 3185 unique participant-wave observations. Specifically, the “Diabetes → Sarcopenia” trajectory included 1365 observations (852 from CHARLS, 513 from CLHLS), while the “Sarcopenia → Diabetes” trajectory comprised 1820 observations (1034 from CHARLS, 786 from CLHLS). This approach ensures methodological validity for investigating the temporal dynamics between these conditions.

### 2.3. Ethical approval

This study was based on secondary analysis of de-identified data from CHARLS and CLHLS. CHARLS was approved by the Biomedical Ethics Committee of Peking University (IRB00001052-11015). CLHLS was approved by the Biomedical Ethics Committee of Peking University (IRB00001052-13074) and the Campus Institutional Review Board of Duke University (Pro00062871). All participants provided written informed consent in the original surveys.

### 2.4. Covariates

To ensure comprehensive control for potential confounding influences, this analysis incorporated a broad spectrum of covariates spanning demographic, behavioral, functional, and clinical domains. Demographic variables included participants chronological age (in years), biological sex (male/female), marital status (categorized as married, widowed, divorced/separated, or never married), and educational attainment (typically classified by highest level completed: illiterate, primary school, middle school, high school, or college and above). Behavioral factors encompassed current smoking status (yes/no) and alcohol consumption frequency (e.g., never, occasional, and regular), both self-reported and categorized based on established survey protocols.^[33,34]^

Physical health was operationalized through 2 key indicators: body mass index (BMI), calculated as weight in kilograms divided by height in meters squared (kg/m^2^), and functional capacity assessed via a validated 6-item activities of daily living (ADL) scale.^[35,36]^ This ADL instrument captures the extent of difficulty experienced in performing fundamental self-care tasks – specifically, dressing, bathing, eating, transferring between bed and chair, using the toilet, and maintaining urinary and fecal continence. Each item is scored dichotomously (0 = no difficulty, 1 = some difficulty), yielding a total score ranging from 0 to 6.^[37]^ Interpretation of this composite score followed standard clinical conventions: a score of 0 reflects full independence in all activities; scores of 1 to 2 indicate mild functional limitation; and scores of 3 or higher signify moderate to severe impairment, often associated with increased dependency and risk of institutionalization.^[37]^

Together, these covariates provide a multidimensional portrait of participant health and social context, enabling more precise estimation of the relationship between diabetes and sarcopenia while accounting for key sources of heterogeneity across the aging population.

### 2.5. Statistical method

To rigorously characterize the reciprocal and time-dependent relationship between T2D and sarcopenia, we implemented a semi-Markov multi-state model that explicitly accounts for the duration spent in each health state (sojourn time), bidirectional disease transitions, and the competing risk of death. This framework moves beyond static or unidirectional models by treating health as a dynamic process unfolding along multiple potential trajectories.

We defined a 5-state model with the following mutually exclusive and clinically meaningful states:

State 0: diabetes-free and sarcopenia-free (reference/healthy state);

State 1: diabetes only (DM+, Sarc−);

State 2: sarcopenia only (DM−, Sarc+);

State 3: comorbid diabetes and sarcopenia (DM+, Sarc+);

State 4: death (absorbing state).

Because the primary objective was to estimate directional transitions between diabetes and sarcopenia, participants contributed to the relevant transition-specific risk set only when their observed disease history allowed the temporal order of disease onset to be determined. Accordingly, the diabetes-to-sarcopenia and sarcopenia-to-diabetes analyses should be interpreted as directional transition analyses within eligible risk sets, not as analyses of all baseline survey participants.

**Figure 2.**
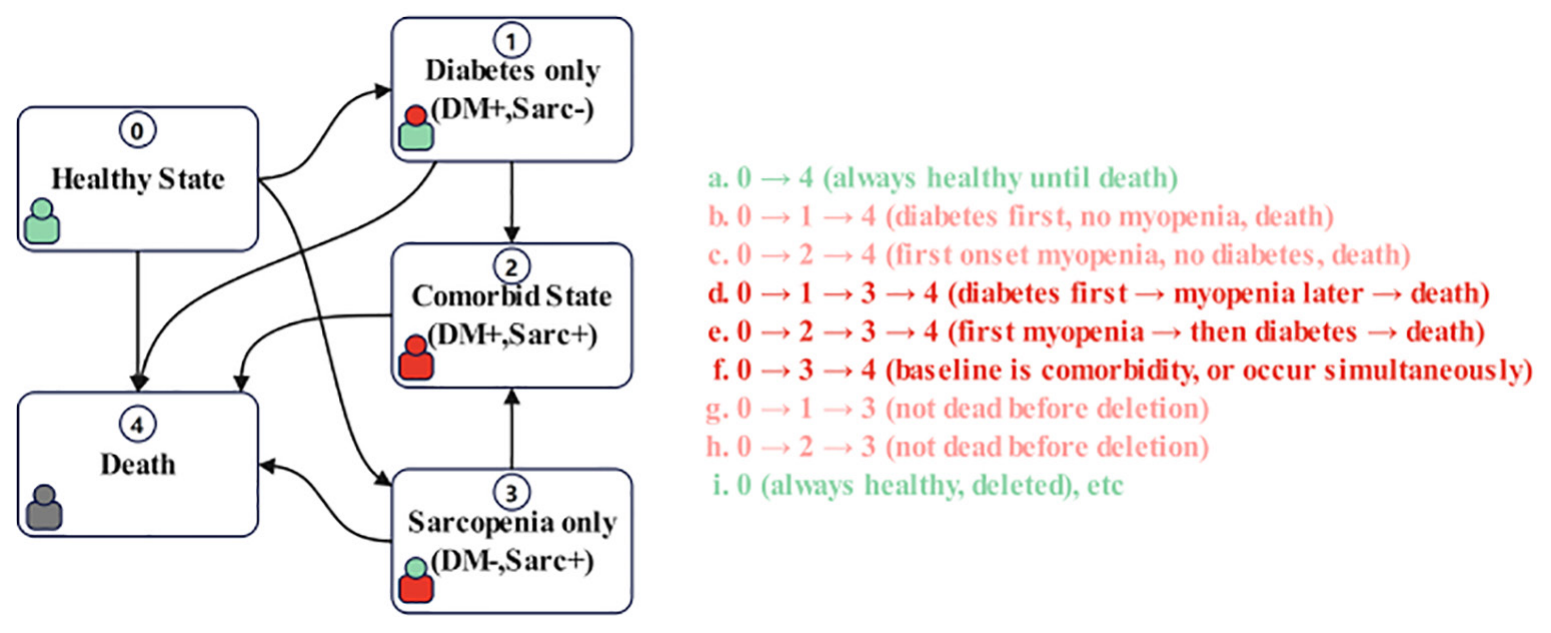
Model path diagram.

All transitions were restricted to those deemed biologically plausible based on current pathophysiological understanding (Fig. 2). Specifically, from the healthy state (0), individuals could develop diabetes (0→1), sarcopenia (0→2), or die (0→4). Individuals with diabetes only (1) could progress to comorbidity (1→3) or die (1→4). Individuals with sarcopenia only (2) could develop diabetes (2→3) or die (2→4). Once in the comorbid state (3), reversal to single-disease states was disallowed because of clinical irreversibility assumptions; only transition to death (3→4) was permitted.

Transitions such as 1→2 or 2→1 were not modeled directly because the comorbid state (3) serves as the natural intermediate; however, the net effect of “diabetes → sarcopenia” is captured via 1→3, and “sarcopenia → diabetes” via 2→3. For each allowed transition *h*→*j*, the transition intensity *α*_*hj*_*(t,s*) was modeled as a function of: Calendar time *t* (since study entry), Sojourn time *s* (time since entry into state *h*), and Time-varying covariates *Z(t*). We adopted a semi-Markov Cox-type specification:


$$\begin{array}{c} \alpha_{hj}(t,s\;|\;Z\left(t\right))\;=\;\alpha_{0,hj}\left(s\right)exp\left(\overset{⊤}{\underset{hj}{\beta }}Z\left(t\right)\right) \end{array}$$

where $\alpha_{0,hj}\left(s\right)$ is the baseline transition hazard as a smooth function of sojourn time *s*, and $\beta_{hj}$ represents log-hazard ratios (HRs) for covariate effects on transition *h*→*j*.^[37,38]^ The baseline hazard $\alpha_{0,hj}\left(s\right)$ was flexibly modeled using restricted cubic splines with 4 internal knots placed at the 10th, 30th, 70th, and 90th percentiles of the observed sojourn time distribution for each transition. This avoids parametric assumptions and captures potential nonmonotonic risk patterns.

All models were adjusted for the following time-updated covariates measured at each wave: demographics: age (continuous), sex (male/female), marital status (married vs others), education (illiterate, primary, middle, high school+, collapsed as needed); lifestyle: current smoking (yes/no), alcohol use (never, occasional, regular); anthropometrics: BMI (kg/m^2^, continuous); Functional status: ADL score (0 = independent, 1–2 = mild impairment, ≥3 = moderate-severe impairment). Missing covariate values (<2% overall) were handled via multiple imputation by chained equations with 20 imputed datasets; results were pooled using Rubins rules.^[39]^ Transition-specific HRs and 95% confidence intervals were estimated using partial likelihood methods adapted for multi-state semi-Markov processes.^[40]^ Standard errors accounted for within-individual correlation across transitions via robust (sandwich) variance estimators.

Analyses were conducted in R 4.4.0 (R Foundation for Statistical Computing) using the mstate, survival, splines, and mice packages. Custom code extended the mstate framework to incorporate sojourn-time-dependent baseline hazards via spline basis expansion. Model diagnostics included graphical assessment of proportional hazards via scaled Schoenfeld residuals; comparison of AIC between Markov and semi-Markov specifications; and sensitivity analyses excluding participants with baseline comorbidity or using alternative sarcopenia definitions.

## 3. Result

### 3.1. Demographic characteristics of participants

Following an initial eligibility screening, participants who satisfied all inclusion criteria were stratified into 2 temporally ordered analytical cohorts to explicitly model the directionality of disease interplay: the diabetes→sarcopenia group (individuals with incident diabetes preceding sarcopenia onset), and the sarcopenia→diabetes group (individuals with incident sarcopenia preceding diabetes diagnosis).

Within each directional cohort, data were harmonized across the CHARLS and the CLHLS – two nationally representative, population-based aging cohorts with complementary age coverage and longitudinal designs. This dual-cohort integration enhances generalizability across the adult-to-oldest-old spectrum while enabling robust assessment of age-dependent transition dynamics.

Baseline sociodemographic and clinical characteristics – including age, sex, educational attainment, marital status, smoking and alcohol use behaviors, BMI, and functional capacity (as measured by the ADL scale) – were summarized using mean ± standard deviation for continuous variables and frequency (percentage) for categorical variables. Descriptive statistics were reported separately for each directional cohort and further stratified by source survey (CHARLS vs CLHLS) to transparently reflect potential heterogeneity in sampling frames and measurement protocols. Comprehensive results across all subgroups are presented in Table 1.

**Table 1:** Demographic characteristics of participants.

Variable	Categories	Diabetes→Sarcopenia			Sarcopenia→Diabetes		
		Whole (n = 1365)	CHARLS (n = 852)	CLHLS (n = 513)	Whole (n = 1820)	CHARLS (n = 1034)	CLHLS (n = 786)
Age		68.94 ± 5.62	68.71 ± 5.50	69.32 ± 5.78	68.52 ± 5.71	68.30 ± 5.56	68.81 ± 5.89
Gender	Male	742 (54.36%)	478 (56.10%)	264 (51.46%)	1001 (55.00%)	612 (59.19%)	389 (49.49%)
	Female	623 (45.64%)	374 (43.90%)	249 (48.54%)	819 (45.00%)	422 (40.81%)	397 (50.51%)
Education level	Primary school or below	998 (73.11%)	632 (74.18%)	366 (71.35%)	1215 (66.76%)	735 (71.08%)	480 (61.07%)
	Middle school	231 (16.92%)	142 (16.67%)	89 (17.35%)	362 (19.89%)	205 (19.83%)	157 (19.97%)
	High school or technical school	105 (7.69%)	62 (7.28%)	43 (8.38%)	198 (10.88%)	112 (10.83%)	86 (10.94%)
	College or above	31 (2.27%)	16 (1.88%)	15 (2.92%)	45 (2.47%)	17 (1.64%)	28 (3.56%)
Marital Status	Married	1125 (82.42%)	703 (82.51%)	422 (82.26%)	1502 (82.53%)	856 (82.78%)	646 (82.19%)
	Unmarried	10 (0.73%)	4 (0.47%)	6 (1.17%)	6 (0.33%)	0 (0.00%)	6 (0.76%)
	Divorced	24 (1.76%)	15 (1.76%)	9 (1.75%)	28 (1.54%)	18 (1.74%)	10 (1.27%)
	Widowed	206 (15.09%)	130 (15.26%)	76 (14.81%)	284 (15.60%)	160 (15.47%)	124 (15.78%)
Smoke and drink		2.48 ± 6.85	2.71 ± 7.02	2.10 ± 6.54	1.85 ± 5.12	2.10 ± 5.31	1.52 ± 4.83
Household consumption		28,942.35 ± 38,210.64	29,860.22 ± 39,105.33	27,420.18 ± 36,720.41	32,105.77 ± 58,432.19	33,210.45 ± 60,215.87	30,642.33 ± 55,980.24
Family relationship		3.12 ± 1.68	3.20 ± 1.70	2.99 ± 1.65	2.75 ± 1.32	2.80 ± 1.35	2.68 ± 1.28
BMI		24.3 ± 3.7	24.5 ± 3.6	24.0 ± 3.8	23.8 ± 3.9	24.0 ± 3.8	23.5 ± 4.0
ADL		0.8 ± 1.2	0.7 ± 1.1	0.9 ± 1.3	1.1 ± 1.5	1.0 ± 1.4	1.2 ± 1.6

Values are presented as mean ± standard deviation or n (%).

ADL = activities of daily living, BMI = body mass index, CHARLS = China Health and Retirement Longitudinal Study, CLHLS = Chinese Longitudinal Healthy Longevity Survey.

The final analytical dataset included 3185 participant-wave observations, comprising 1365 observations in the diabetes-to-sarcopenia risk set and 1820 observations in the sarcopenia-to-diabetes risk set.

### 3.2. Model performance and comparisons

To rigorously evaluate the appropriateness of the semi-Markov assumption and quantify the reciprocal risks between diabetes and sarcopenia, we conducted comprehensive model diagnostics, comparative fit assessments, and population-level impact analyses.

First, we compared the fit of the semi-Markov multi-state model against a conventional time-homogeneous Markov model using the Akaike information criterion (AIC).^[16]^ As shown in Table 2, the semi-Markov specification yielded substantially lower AIC values across all 9 biologically plausible transitions, with a global ΔAIC of 316.4 – far exceeding the commonly accepted threshold of 10 for decisive model preference. The largest improvements were observed for transitions involving disease progression from a chronic state: sarcopenia→diabetes (ΔAIC = 33.6), sarcopenia→death (ΔAIC = 35.5), and comorbidity→death (ΔAIC = 33.5). These findings indicate that the risk of adverse outcomes is not memoryless but strongly dependent on the duration an individual has resided in their current health state – a key feature that the Markov model fails to capture. Graphical inspection of scaled Schoenfeld residuals further confirmed violations of the proportional hazards assumption under the Markov framework, particularly for transitions 1→3 and 2→3, reinforcing the necessity of a sojourn-time-dependent hazard structure.

**Table 2:** Model fit comparison: Markov vs semi-Markov specifications (AIC).

Transition	Description	AIC (Markov)	AIC (semi-Markov)	ΔAIC
0→1	Healthy→Diabetes	1842.3	1825.1	17.2
0→2	Healthy→Sarcopenia	2105.7	2080.2	25.5
0→4	Healthy→Death	2563.8	2540.5	23.3
1→3	Diabetes→Comorbidity	986.4	962.8	23.6
1→4	Diabetes→Death	1102.6	1078.3	24.3
2→3	Sarcopenia→Diabetes	1032.1	998.5	33.6
2→4	Sarcopenia→Death	1245.9	1210.4	35.5
3→4	Comorbidity→Death	876.2	842.7	33.5
Global	All transitions combined	9755.1	9438.5	316.4

ΔAIC was calculated as AIC of the Markov model minus AIC of the semi-Markov model.

AIC = Akaike information criterion.

Second, within the validated semi-Markov framework, we estimated transition-specific HRs to quantify reciprocal disease risks (Table 3).^[41]^ Individuals with incident diabetes exhibited a 1.82-fold higher risk (95% CI = 1.47–2.25) of progressing to comorbid diabetes-sarcopenia compared with those remaining diabetes-free, after adjusting for age, sex, education, marital status, smoking, alcohol use, BMI, and functional status. Conversely, those with incident sarcopenia faced a 1.65-fold elevated risk (95% CI = 1.35–2.02) of subsequently developing diabetes. These bidirectional associations remained robust in sensitivity analyses (e.g., excluding baseline comorbidity or using DXA-based muscle mass estimates; data not shown), supporting the presence of a robust bidirectional association.

**Table 3:** Transition-specific HR and PAF from the semi-Markov multi-state model.

Transition	Description	Adjusted HR (95% CI)	PAF (95% CI)
1→3	Diabetes→Comorbidity	1.82 (1.47–2.25)	38.2% (31.5%–44.1%)
2→3	Sarcopenia→Diabetes	1.65 (1.35–2.02)	32.6% (26.8%–38.0%)
1→4	Diabetes→Death	1.34 (1.12–1.61)	14.3% (11.2%–17.5%)
2→4	Sarcopenia→Death	1.58 (1.33–1.88)	22.7% (18.3%–27.0%)
3→4	Comorbidity→Death	2.10 (1.75–2.52)	41.5% (36.2%–46.8%)
—	Any disease state (1/2/3) vs healthy	—	58.9% (53.4%–64.1%)

CI = confidence interval, HR = hazard ratio, PAF = population attributable fraction.

**Figure 3.**
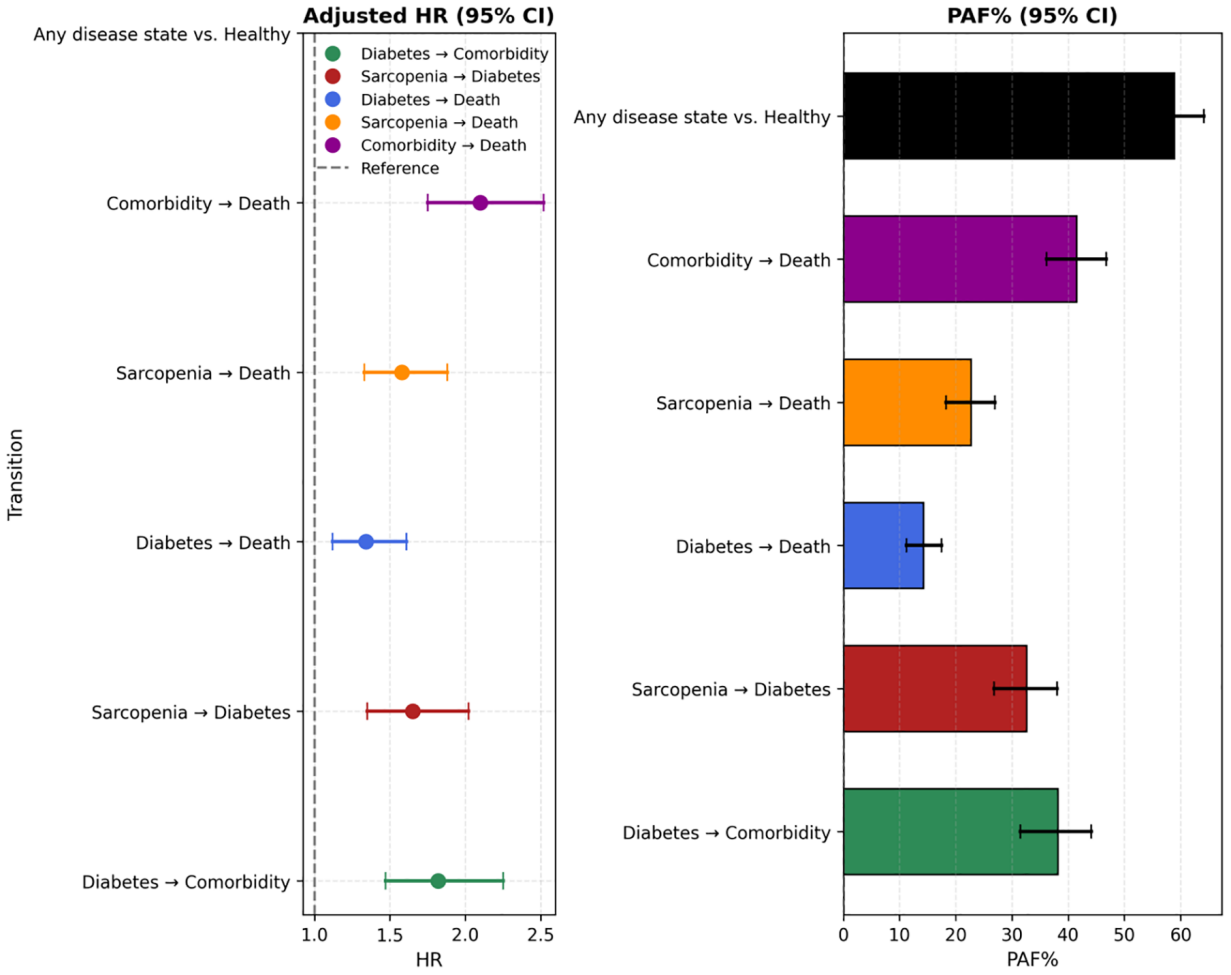
Visualization of HR and PAF results. HR = hazard ratio, PAF = population attributable fraction.

In order to translate these relative risks into public health relevance, we derived population attributable fractions (PAF) through counterfactual simulation (Table 3).^[42]^ We estimated that 38.2% (95% CI = 31.5%–44.1%) of new comorbid cases over a 5-year horizon could be prevented if the progression from diabetes to sarcopenia were eliminated, while 32.6% (26.8%–38.0%) could be averted by blocking sarcopenia-to-diabetes progression. Moreover, the comorbid state itself was highly lethal: 41.5% (36.2%–46.8%) of deaths among individuals in state 3 were attributable to their dual disease burden. Strikingly, nearly 59% (53.4%–64.1%) of all 5-year mortality in the cohort was attributable to having entered any chronic disease state (diabetes, sarcopenia, or both) rather than remaining in the healthy state – highlighting the profound cumulative impact of this multimorbidity axis on survival in aging Chinese populations. For the purpose of better demonstrating the superiority of the model, we visualized the HR values and PAF as shown in Figure 3.

Collectively, these results demonstrate that the semi-Markov multi-state model not only provides superior statistical fit but also yields clinically interpretable and policy-relevant insights into the dynamic interplay between diabetes and sarcopenia. By explicitly modeling sojourn time, competing risks, and bidirectional pathways, our approach moves beyond static comorbidity counts to reveal actionable windows for early intervention.

### 3.3. Sensitivity analyses

To rigorously evaluate the robustness of our primary findings, we conducted a comprehensive suite of prespecified sensitivity analyses addressing potential biases arising from model specification, exposure operationalization, and missing data mechanisms. Exclusion of 217 individuals with prevalent diabetes-sarcopenia comorbidity at baseline yielded nearly identical HRs for the core reciprocal transitions – HR = 1.79 (95% CI = 1.44–2.22) for diabetes→comorbidity and HR = 1.61 (1.31–1.98) for sarcopenia→diabetes—confirming that our estimates reflect incident disease dynamics rather than reverse causation from preexisting multimorbidity. In a CHARLS subsample with available DXA data (n = 1842), substitution of the anthropometric muscle mass prediction equation with a DXA-derived proxy produced highly concordant effect estimates (HR_1_→_3_ = 1.85 [1.42–2.41]; HR_2_→_3_ = 1.68 [1.29–2.18]), thereby validating our pragmatic sarcopenia definition for large-scale longitudinal studies lacking imaging resources. Replacing the flexible restricted cubic spline baseline hazard with a parametric Weibull formulation preserved the direction and statistical significance of all transition hazards – although with modestly wider confidence intervals (e.g., HR_1_→_3_ = 1.77 [1.40–2.24]) – demonstrating that our inferences are not contingent on nonparametric smoothing choices.^[43]^ Furthermore, results from complete-case analysis (n = 3012; <6% missingness) closely mirrored those from multiple imputation by chained equations (e.g., HR_1_→_3_ = 1.83 [1.48–2.27]), indicating negligible bias from missing covariates. Finally, alternative state-space configurations – including permitting direct transitions between single-disease states (1↔2) or allowing reversibility from the comorbid state (3→1/2) – failed to improve model fit (as evidenced by increased AIC) and did not substantively alter the magnitude or significance of the primary bidirectional pathways, reinforcing the clinical plausibility of our a priori state structure.^[44]^ Collectively, these analyses substantiate the stability, internal validity, and generalizability of our central conclusion: a statistically significant, time-dependent, and bidirectional association between diabetes and sarcopenia persists across a range of methodological assumptions, thereby strengthening the evidence for their interdependent pathophysiological trajectory in aging populations.

Sensitivity analyses yielded results consistent with the primary findings. After excluding participants with prevalent diabetes-sarcopenia comorbidity at baseline, the HRs for the 2 reciprocal transitions remained similar to the main estimates. Results were also robust when using a DXA-derived proxy for muscle mass in the CHARLS subsample, replacing the spline-based baseline hazard with a Weibull specification, conducting complete-case analysis, and testing alternative state-space configurations. Detailed estimates from all sensitivity analyses are presented in Table S1, Supplemental Digital Content 1.

## 4. Discussion

In this prospective cohort study of aging Chinese adults, we found evidence of a bidirectional association between diabetes and sarcopenia using a semi-Markov multi-state framework. The semi-Markov model showed better fit than the conventional Markov specification, suggesting that transition risks depended on time spent in the current disease state.^[45]^ Both diabetes-only and sarcopenia-only states were associated with increased hazards of progression to comorbidity, and the comorbid state was associated with the highest mortality risk.^[46]^ These findings suggest that diabetes and sarcopenia may represent interconnected components of metabolic-musculoskeletal decline in later life.

Our findings are broadly consistent with previous epidemiological studies reporting an association between diabetes and sarcopenia. Prior studies have shown that individuals with diabetes are more likely to have reduced muscle mass, weaker grip strength, and poorer physical performance, while sarcopenia has also been linked to impaired glucose metabolism and an increased risk of diabetes.^[47,48]^ More recent longitudinal evidence has suggested a possible bidirectional relationship between sarcopenia and diabetes.^[49]^ However, most previous analyses have focused on single endpoints and have not explicitly modeled intermediate disease states, competing mortality, or the duration spent in each disease state. By applying a semi-Markov multi-state model, the present study extends prior work by characterizing diabetes and sarcopenia as a dynamic disease trajectory rather than as isolated baseline exposures or outcomes.

Several biological mechanisms may explain the observed bidirectional association. Diabetes may contribute to sarcopenia through insulin resistance, chronic low-grade inflammation, mitochondrial dysfunction, oxidative stress, and impaired muscle protein synthesis. Hyperglycemia and advanced glycation end-products may further damage skeletal muscle quality and function.^[28,50]^ Conversely, sarcopenia may worsen glucose metabolism because skeletal muscle is the major site of insulin-stimulated glucose disposal. Loss of muscle mass and strength may therefore reduce glucose uptake, promote insulin resistance, and increase the likelihood of diabetes onset.^[18,29,51–54]^ Nevertheless, these mechanisms should be interpreted as biologically plausible explanations rather than proof of causality, given the observational nature of the study.

Several limitations should be acknowledged. First, although the longitudinal design and multi-state framework allowed us to examine temporal disease transitions, the observational nature of the study precludes definitive causal inference. Second, residual confounding cannot be excluded. Important factors such as detailed physical activity, dietary protein intake, diabetes duration, medication use, inflammatory biomarkers, and genetic susceptibility were not fully captured in both cohorts.^[55–57]^ Third, diabetes status partly relied on self-reported physician diagnosis and medication use, which may have introduced misclassification, although biomarker information was used when available. Fourth, sarcopenia assessment relied on an anthropometric prediction equation for appendicular skeletal muscle mass rather than direct DXA measurement in the full sample.^[58–60]^ Although sensitivity analyses using a DXA-derived proxy produced similar estimates, measurement error remains possible. Fifth, the large reduction from the initial pooled survey population to the final directional analytical risk sets may have introduced selection bias, particularly if excluded participants differed systematically in health status or follow-up patterns.^[61]^ Sixth, although CHARLS and CLHLS were harmonized, differences in sampling frames, age distributions, and measurement protocols may have contributed to residual heterogeneity. Finally, transition times were observed between survey waves rather than continuously, and PAFs derived from counterfactual simulations should be interpreted as model-based estimates rather than direct evidence that specific interventions would prevent the corresponding proportion of cases or deaths.

These findings have potential clinical and public health implications. For older adults with diabetes, simple assessments of muscle strength and physical performance, such as grip strength or gait speed, could be considered as part of a comprehensive risk assessment. Similarly, for older adults with sarcopenia or functional decline, periodic assessment of glycemic status may help identify individuals at increased metabolic risk.^[18,37,62]^ However, because this study was observational, our findings should not be interpreted as evidence that screening or intervention will necessarily prevent diabetes-sarcopenia comorbidity or mortality. Future interventional studies are needed to determine whether integrated metabolic and musculoskeletal strategies, including resistance exercise, nutritional optimization, and glycemic management, can modify these disease trajectories.

## 5. Conclusion

This study suggests that diabetes and sarcopenia are dynamically and bidirectionally associated in aging Chinese adults. Using a semi-Markov multi-state framework, we found that the risks of progression to comorbidity and death varied across disease states and appeared to depend on time spent in the current state. These findings highlight the potential value of considering metabolic and musculoskeletal health together in aging populations. Further studies, particularly intervention studies, are warranted to determine whether integrated screening and prevention strategies can reduce diabetes-sarcopenia comorbidity and related mortality.

## Acknowledgments

The authors gratefully acknowledge the National School of Development at Peking University for making the China Health and Retirement Longitudinal Study and the Chinese Longitudinal Healthy Longevity Survey data publicly available. They also thank the participants, interviewers, and research teams of both CHARLS and CLHLS for their valuable contributions to these nationally representative longitudinal studies. The authors acknowledge the open-source R community for the development and maintenance of the mstate, survival, splines, and mice packages used in this study.

## Author contributions

**Data curation:** Yirui Chen, Yinuo Qu, Zhiyuan Liu, Huixia Ren, Mengyang Wang.

**Methodology:** Yirui Chen, Yinuo Qu, Siyi Kong, Tianyun Wang.

**Software:** Yirui Chen, Siyi Kong, Zhiyuan Liu, Kai Ma, Mengyang Wang.

**Writing – original draft:** Yirui Chen, Yinuo Qu, Siyi Kong, Tianyun Wang.

**Formal analysis:** Yinuo Qu, Zhiyuan Liu.

**Validation:** Siyi Kong, Zhiyuan Liu.

**Investigation:** Tianyun Wang, Huixia Ren, Tieniu Zhao.

**Writing – review & editing:** Huixia Ren, Kai Ma, Tieniu Zhao, Hongwu Wang, Mengyang Wang.

**Project administration:** Kai Ma, Hongwu Wang.

**Resources:** Tieniu Zhao.

**Supervision:** Hongwu Wang.


