# Supplementary material for: Reciprocal risks of diabetes and sarcopenia in aging Chinese adults: A prospective cohort study using a semi-Markov multi-state framework
Source: Medicine (Baltimore). 2026 Jun 26;105(26):e49398. doi: 10.1097/MD.0000000000049398 (PMC13313702; doi:10.1097/MD.0000000000049398)
Supplement: Supplementary file 1 [file medi-105-e49398-s001.docx]

**Supplementary Table S1. Sensitivity analyses for the reciprocal transitions between diabetes and sarcopenia**

| Analysis | Sample / model specification | Diabetes → Comorbidity, 1→3 Adjusted HR (95% CI) | Sarcopenia → Diabetes, 2→3 Adjusted HR (95% CI) | Interpretation |
| --- | --- | --- | --- | --- |
| Primary analysis | Semi-Markov multi-state model; multiple imputation; full analytical risk sets | 1.82 (1.47–2.25) | 1.65 (1.35–2.02) | Primary result |
| Excluding prevalent comorbidity at baseline | Excluded 217 participants with diabetes-sarcopenia comorbidity at baseline | 1.79 (1.44–2.22) | 1.61 (1.31–1.98) | Estimates remained similar to primary analysis |
| Alternative sarcopenia definition using DXA-derived proxy | CHARLS subsample with available DXA-derived muscle mass proxy, n = 1,842 | 1.85 (1.42–2.41) | 1.68 (1.29–2.18) | Results were robust to alternative muscle mass assessment |
| Parametric Weibull baseline hazard | Semi-Markov model with Weibull baseline hazard instead of restricted cubic splines | 1.77 (1.40–2.24) | 1.62 (1.27–2.06) | Direction and statistical significance were preserved |
| Complete-case analysis | Participants with complete covariate data, n = 3,012 | 1.83 (1.48–2.27) | 1.66 (1.34–2.05) | Results were consistent with multiple-imputation analysis |
| Alternative state-space model I | Additional direct transitions between single-disease states allowed, 1↔2 | 1.80 (1.45–2.23) | 1.63 (1.32–2.01) | No substantive change in reciprocal transition estimates |
| Alternative state-space model II | Reversibility from comorbid state allowed, 3→1 or 3→2 | 1.78 (1.43–2.21) | 1.62 (1.30–2.02) | Model fit did not improve; primary conclusions unchanged |
| Alternative state-space model III | Fully extended model allowing both 1↔2 and 3→1/2 transitions | 1.79 (1.44–2.22) | 1.64 (1.32–2.04) | Findings remained stable under a more flexible transition structure |

Note: Values are adjusted hazard ratios and 95% confidence intervals. All models were adjusted for age, sex, marital status, education, smoking, alcohol use, body mass index, and activities of daily living score. CI, confidence interval; DXA, dual-energy X-ray absorptiometry; HR, hazard ratio.
